# Weighing Successes and Challenges in the Establishment of Additional Epilepsy Monitoring Units at the Saudi Arabian Kingdom's Capital City

**DOI:** 10.7759/cureus.38924

**Published:** 2023-05-12

**Authors:** Alawi A Al-Attas

**Affiliations:** 1 Epilepsy, King Saud Medical City, Riyadh, SAU

**Keywords:** presurgical evaluation, arabian peninsula, saudi arabia, epilepsy monitoring unit, saudi healthcare systems

## Abstract

Epilepsy has a prevalence rate of 6.54 per 1,000 people in Saudi Arabia, making it a prevalent chronic condition. Drug-resistant epilepsy (DRE) is thought to affect one-third of patients; in these circumstances, a complete presurgical examination in the epilepsy monitoring unit (EMU) is necessary. Unfortunately, to accommodate the growing number of referrals, the units' availability and number must be reviewed.

## Editorial

With a projected population of 36,850,914 in 2023, the Kingdom of Saudi Arabia is the largest nation on the Arabian Peninsula and one of the most influential nations in the entire globe. In addition, the population of Riyadh, the capital and largest city of Saudi Arabia, was 7,682,430 as of the 2023 census. Riyadh's population has increased by 144,230 (or 1.91% yearly) over the last year, even though there were only 111,123 individuals in the city in 1950, with a strong possibility of exceeding 9,058,394 by 2035 [1].

An epilepsy monitoring unit (EMU) is a specialized unit intended to monitor patients for presurgical evaluation or differentiate between true epilepsy and psychogenic non-epileptic events. EMU facilitates monitoring and treating patients with drug-resistant epilepsy (DRE). There are only 11 EMUs in Saudi Arabia, according to Aljafen et al. [2], and only four eminent government hospitals in Riyadh have well-established EMUs with approximately 5-6 beds per unit. Given the number of hospitals that have been converted into medical cities, there is a good likelihood that there will be more EMUs that are well-organized and structured. King Saud Medical City (KSMC) is a center of excellence for educational training and one of the major cities in the kingdom that offers top-notch healthcare to patients. It is also a regional leader in the use of technology, medical reference laboratory services, and innovation in these areas. Indeed, the neurology department, with strong administrative support, has established epilepsy and neurophysiology services with an ambitious pursuit to establish a well-structured EMU in the near future.

The Health Sector Transformation Program aims to restructure the Saudi Arabian healthcare system to become a comprehensive, efficient, and integrated health system that is based on the health of the individual and society (including the citizen, the resident, and the visitor) to meet the Kingdom's Vision 2030 and ensure the continued development of healthcare services in Saudi Arabia. Along with aligning and connecting with strategic national goals during the transformation process, the Health Sector Transformation Program also aims to harmonize and coordinate with all entities in the health sector, Vision Realization Programs (VRPs), and other government bodies [3]. The strategic plan is to establish more large, specialized units, including EMUs, to provide this service to all epileptic patients; promote healthcare in Saudi Arabia, the Gulf, and Arab countries; and share our experience to support such units shortly.

Determine the main problem

Epilepsy is the most common neurological disorder after headache and stroke in this country, with a prevalence of 6.54 per 1,000 people. Approximately seven in every 1,000 Saudis have epilepsy. Moreover, epilepsy affects daily living activities such as driving and deprives patients of education and employment due to recurrent seizures, which increase the risk of mortality such as sudden unexpected death from epilepsy (SUDEP) and morbidity rates if untreated properly. Furthermore, seizures lead to personal embarrassment and loss of dignity, stigma, discrimination, social exclusion, transportation problems, and a variety of psychosocial difficulties. In fact, there are only four well-established EMUs in Riyadh City, with capacity ranging from three to five beds in each unit, a 12- to 15-bed occupancy rate, and approximately 400-500 patients per year monitored. Hence, at least three to four more EMUs are needed in Riyadh City, with four to five beds each or even more, to meet the needs and demands of patients with epilepsy (PWE) in Saudi Arabia. Moreover, EMUs in Saudi Arabia were underutilized considering the number of admitted patients and the number of epilepsy surgeries per year due to the low potential to establish EMUs in most hospitals, with significant challenges being a shortage of adult and pediatric epileptologists [2,4]. The strategic plan is to establish more EMUs in Saudi Arabia to promote health and quality of life, provide therapeutic value to patients, and increase productivity. Thus, it enhances care for many PWE who have no access to such services. The main goal is to construct the most significant and influential EMUs in Arab nations. However, the specific objectives are to assess DRE cases; lessen the burden of epilepsy on patients and their caregivers; remove the stigma associated with epilepsy by educating patients and their families about it; raise awareness of epilepsy and its management among patients, caregivers, the public, and nonspecialized healthcare providers; and provide accessible and acceptable service, that is, taking sociocultural factors into consideration. All adult and child epileptic patients, regardless of gender or age, who have DRE, defined as the inability to take two properly and carefully selected antiepileptic medications, will have access to this service [5]. PWE who fit the aforementioned criteria will have unrestricted access to our service. As the number of epilepsy cases rises, we may expand our coverage to include some cases from the Gulf countries and other regions. These units will cover patients from various healthcare services.

Challenges

The Ministry of Health must authorize and provide funding for the units, which could take some time. They also lack personnel resources, particularly epileptologists, qualified nurses, and electroencephalography (EEG) technologists. It can be challenging to allot a separate ward with single beds because it is intended for the privacy and security of the patients. Another challenge is to create policies and guidelines or abide by them, as the current epilepsy center guidelines may conflict with hospital policy.

EMU requirements

EMU requires qualified adult and/or pediatric epileptologists, one to two epilepsy neurosurgeons, well-trained EEG technologists, neuropsychologists, and neuroradiologists. Moreover, highly qualified nurses are also needed.

Advantages of EMU

By adopting innovative technology, the presence of such units will allow us to use early diagnosis and management of refractory epilepsy, lessen the burden on other neurology services, improve the level and quality of healthcare services, and establish ourselves as the leading unit in the Arab world in that field.

In conclusion, EMU services are still underutilized in Saudi Arabia, despite the country's excellent healthcare system. According to speculation, the Kingdom's Vision 2030 and the nation's present healthcare system change will lead to a rise in the number of these units.
